# Poor response to artesunate treatment in two patients with severe malaria on the Thai–Myanmar border

**DOI:** 10.1186/s12936-018-2182-z

**Published:** 2018-01-15

**Authors:** Aung Pyae Phyo, Kyaw Kyaw Win, Aung Myint Thu, Lei Lei Swe, Htike Htike, Candy Beau, Kanlaya Sriprawat, Markus Winterberg, Stephane Proux, Mallika Imwong, Elizabeth A. Ashley, Francois Nosten

**Affiliations:** 1 Shoklo Malaria Research Unit, Mahidol-Oxford Tropical Medicine Research Unit, Faculty of Tropical Medicine, Mahidol University, PO Box 46, Mae Sot, Tak, 63110 Thailand; 2 Mahidol-Oxford Tropical Medicine Research Unit, Faculty of Tropical Medicine, Mahidol University, Bangkok, Thailand; 3 Department of Molecular Tropical Medicine and Genetics, Faculty of Tropical Medicine, Mahidol University, Bangkok, Thailand; 4 Centre for Tropical Medicine and Global Health, Nuffield Department of Medicine, University of Oxford, Oxford, UK; 5 Mae Tao Clinic, 702, Moo 1, Tha Sai Luad, Mae Sot, Tak, 63110 Thailand; 6 Myanmar Oxford Clinical Research Unit, Yangon, Myanmar

**Keywords:** Severe malaria, Artemisinin resistance, Quinine, Artesunate, Kelch 13

## Abstract

**Background:**

Malaria has declined dramatically along the Thai–Myanmar border in recent years due to malaria control and elimination programmes. However, at the same time, artemisinin resistance has spread, raising concerns about the efficacy of parenteral artesunate for the treatment of severe malaria.

**Case presentation:**

In November 2015 and April 2017, two patients were treated for severe malaria with parenteral artesunate. Quinine was added within 24 h due to an initial poor response to treatment. The first patient died within 24 h of starting treatment and the second did not clear his peripheral parasitaemia until 11 days later. Genotyping revealed artemisinin resistance Kelch-13 markers.

**Conclusions:**

Reliable efficacy of artesunate for the treatment of severe malaria may no longer be assured in areas where artemisinin resistance has emerged. Empirical addition of parenteral quinine to artesunate for treatment is recommended as a precautionary measure.

**Electronic supplementary material:**

The online version of this article (10.1186/s12936-018-2182-z) contains supplementary material, which is available to authorized users.

## Background

Parenteral artesunate and quinine are the recommended first-line drugs for the treatment of severe *Plasmodium falciparum* malaria [1]. Artesunate has a broader stage-specificity of action than quinine [2] and as a result acts more rapidly, i.e., artesunate kills circulating ring-stage parasites, which are later cleared by the spleen, whereas quinine does not. Evidence from multi-centre clinical trials of patients with severe malaria in Southeast Asia [3] and Africa [4] have shown a significant mortality reduction following artesunate treatment compared to quinine in adults and children.

However, recent emergence of artemisinin resistance characterized by a slow parasite clearance is raising the possibility of erosion of the life-saving properties of the artemisinin derivatives when treating patients with severe malaria, although it has not been possible to study this under trial conditions due to the low number of cases. There were only four cases of severe malaria hospitalized in Shoklo Malaria Research Unit between 2015 to date compared to 38 cases in 2011 [5]. Here, two cases of severe malaria with a poor response to parenteral artesunate are presented.

## Case presentations

### Case 1: At the village

A 30-year-old male farmer with no past history of malaria presented to a malaria post close to the Myanmar–Thai border in November 2015, with 3 days of fever and headache. He showed no signs of severity and was found to be positive for *P. falciparum* with an HRP-2-based rapid diagnostic test. He was treated with oral artemether–lumefantrine by the village malaria post (first dose supervised). The next morning, he returned complaining of anuria, when the village malaria worker referred him to a clinic in Thailand.

### At the clinic

On arrival, the patient appeared slightly jaundiced, was apyrexial and fully conscious. The blood smear revealed a high *P. falciparum* asexual parasitaemia of 20% of infected red blood cells (iRBC) or 757,368 µL. The presence of schizonts (352 µL) and gametocytes (1216 µL) was noted, as well as malaria pigment in the neutrophils. Capillary blood haematocrit (Hct) was 30%, blood glucose 82 mg%, pulse 88 beats/min, respiratory rate 32/min, SPO_2_ 98% in air, blood pressure 110/60 mmHg. His liver was palpable, 3 cm below the costal margin and his spleen was not palpable. Physical examination was otherwise unremarkable. The patient was treated with intravenous artesunate (2.4 mg/kg) because of the oligo-anuria and high parasitaemia [6] and rehydrated with 2.5 L of normal saline over 6 h. His vital signs, blood glucose, conscious level and parasitaemia were monitored 4-hourly and these remained within normal limits.

**Fig. 1:**
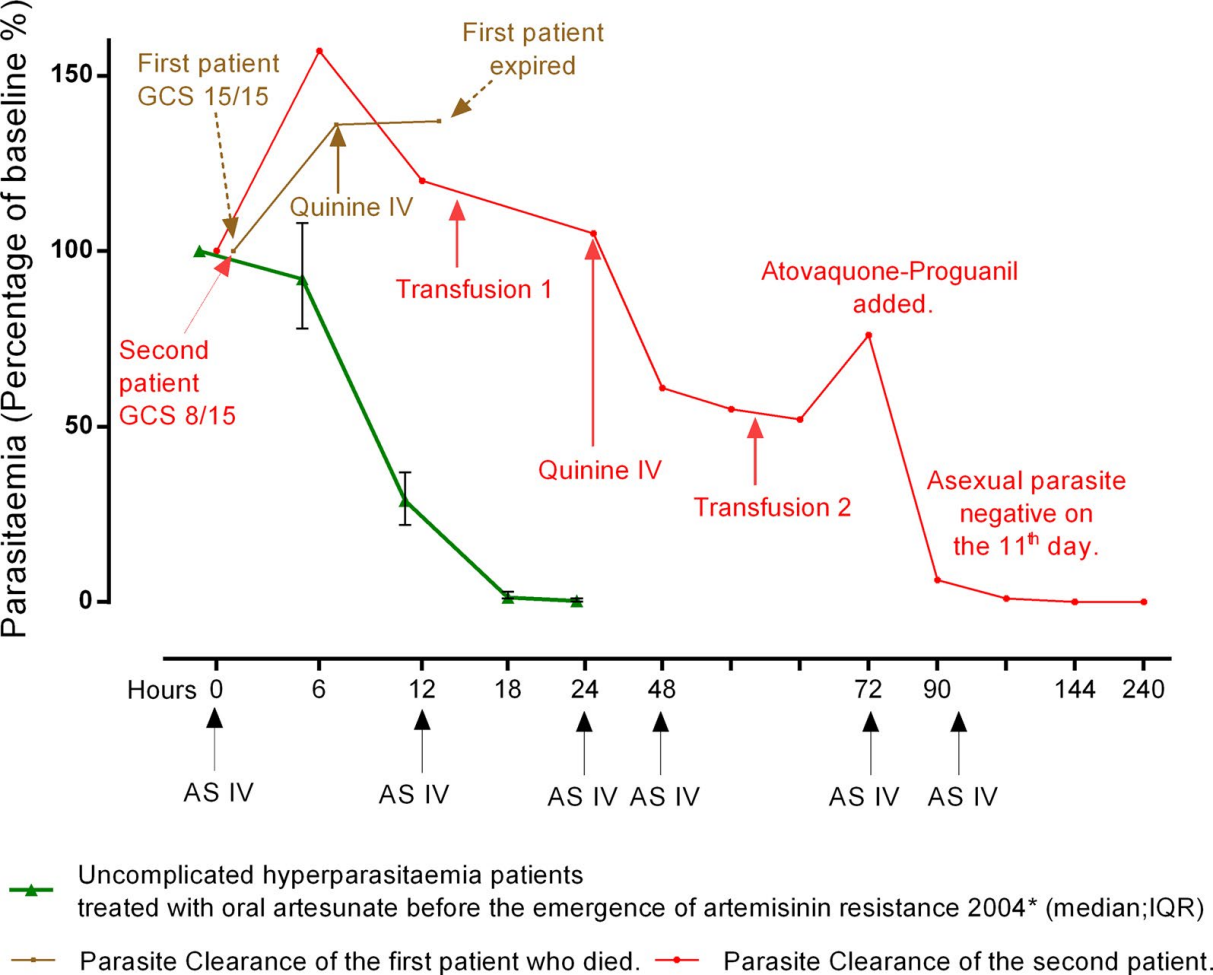
Clinical and parasitological response of two severe malaria cases compared to those patients with uncomplicated hyperparasitaemia who received artesunate per oral (green) (median: IQR)

However, the patient remained oligo-anuric and was referred to another clinic equipped with a biochemistry analyser. Blood urea nitrogen [BUN] (normal range) was 91.8 (2.5–7.1 mmol/L), creatinine 2.5 (0.67–1.17 mg/dL), potassium 4.47 (3.5–5.1 mmol/L), sodium 135.8 (136–145 mmol/L), bicarbonate 4.9 (23–29 mmol/L), blood glucose was 74 mg/dL and Hct dropped to 22%, so 350 mL whole blood was transfused. His Glasgow Coma Score (GCS) was 15/15; he was eating and walking unsupported, had a respiratory rate of 40/min, clear lungs and a temperature of 36.9 °C, blood pressure of 90/40 mmHg. His asexual parasitaemia was now 27% of iRBC or 746,064 µL (> 80% of early ring stage), gametocytaemia 1824 µL, schizontaemia 32 µL. A loading dose (20 mg/kg) of quinine 4-hourly infusion was added. Four hours after the start of the loading dose quinine infusion (quinine in 500 mL of dextrose 10%) the patient’s condition was unchanged and his asexual parasitaemia was now 26% iRBC or 718,432 µL (> 80% early ring stage), gametocytaemia 1920 µL, schizontaemia 16 µL (Fig. 1), blood glucose was 104 mg/dL. A second dose of intravenous artesunate 2.4 mg/kg was given. After 4.5 L of fluid (including fluid bolus) the patient passed 60 mL of high-coloured urine and his temperature rose to 39.7 °C. Fourteen hours after arrival, the patient became unconscious, had a generalized seizure followed by cardiac arrest. Resuscitation was unsuccessful.

### Case 2

On 3 April, 2017, an 18 years old male was admitted with cerebral malaria: *P. falciparum* asexual parasitaemia 38% iRBC or 1,050,016 µL (late stage trophozoite 40%). HIV rapid test was positive, which was confirmed subsequently with a baseline CD4 count of 39 cells/mL. The patient’s GCS was 8/15, tympanic temperature 39.0 °C, blood glucose 51 mg/dL, and blood pressure 120/80 mmHg. His liver was palpable, 3 cm below the costal margin and his spleen was not palpable, although confirmed to be present on abdominal ultrasonography. Intravenous artesunate (2.4 mg/kg) was given on admission, after 12 h and again after 24 h as per protocol [1], i.e., three doses within the first 24 h. However, 24 h after the start of treatment, the parasitaemia was still 34% of iRBC or 1,069,107 µL (> 90% late trophozoites) and a loading dose of quinine (20 mg/kg) was added. At hour 24, he received a blood transfusion of 350 mL whole blood.

After 72 h, the patient recovered consciousness and started eating. However, the parasitaemia had increased again (Fig. 1). Atovaquone–proguanil combination was given in addition to the intravenous artesunate and quinine. By hour-90, the asexual parasitaemia was 7% iRBC or 200,221 µL and he received a second transfusion. Intravenous artesunate and quinine infusion were given for 7 days in total. By day 10 his malaria slide was negative. The patient was discharged on day 12 of hospital admission.

### Anti-malarial drug quality

The anti-malarials used were artemether-lumefantrine (Macleods Pharmaceuticals: EAB 5412B) and artesunate injection (Atlantic pharma: LA170333) provided by the Global Fund malaria programme. Quinine injection (A.N.B laboratories Co. Ltd: 555009) and atovaquone–proguanil (Malarone GSK: GS 0009 Dec 2018) were purchased from the supplier. All the drugs were in date.

### Laboratory tests

All blood slides were double-checked by an expert microscopist of Shoklo Malaria Research Unit.

### Parasite genotyping

Leftover blood from the patients was sent for parasite Kelch-13 genotyping with patient or relative written consent. Both had kelch mutant parasites: patient 1 with the P441L mutation and patient 2 the C580Y mutation. Microsatellite genotyping of flanking regions of C580Y haplotype of the Kelch-13 gene revealed a different C580Y lineage from those described recently in Western Cambodia/Northeastern Thailand/Southern Laos/Myanmar, suggesting a different parental origin [7].

### Plasma drug concentrations

In Case 1, artesunate or DHA were not detected which was not unexpected since the blood sampling time was ~ 24 h after the second (last) dose of artemether-lumefantrine. However, lumefantrine was also undetectable, which could be due to poor absorption of drug since the tablets were taken with water [8] or non-adherence since the second dose was not supervised.

In Case 2, the plasma concentration of artesunate after ~ 2 h of intravenous injection was 105 ng/mL and DHA was 1530 ng/mL, which were well above the plasma levels providing maximum parasiticidal effect [9].

### In vitro parasite culture

Parasite isolates taken from patient 2, after two doses of intravenous artesunate injection (~ 2 h of second dose) grew normally in drug free plate (Additional file 1).

### In vitro sensitivity assay

**Fig. 2:**
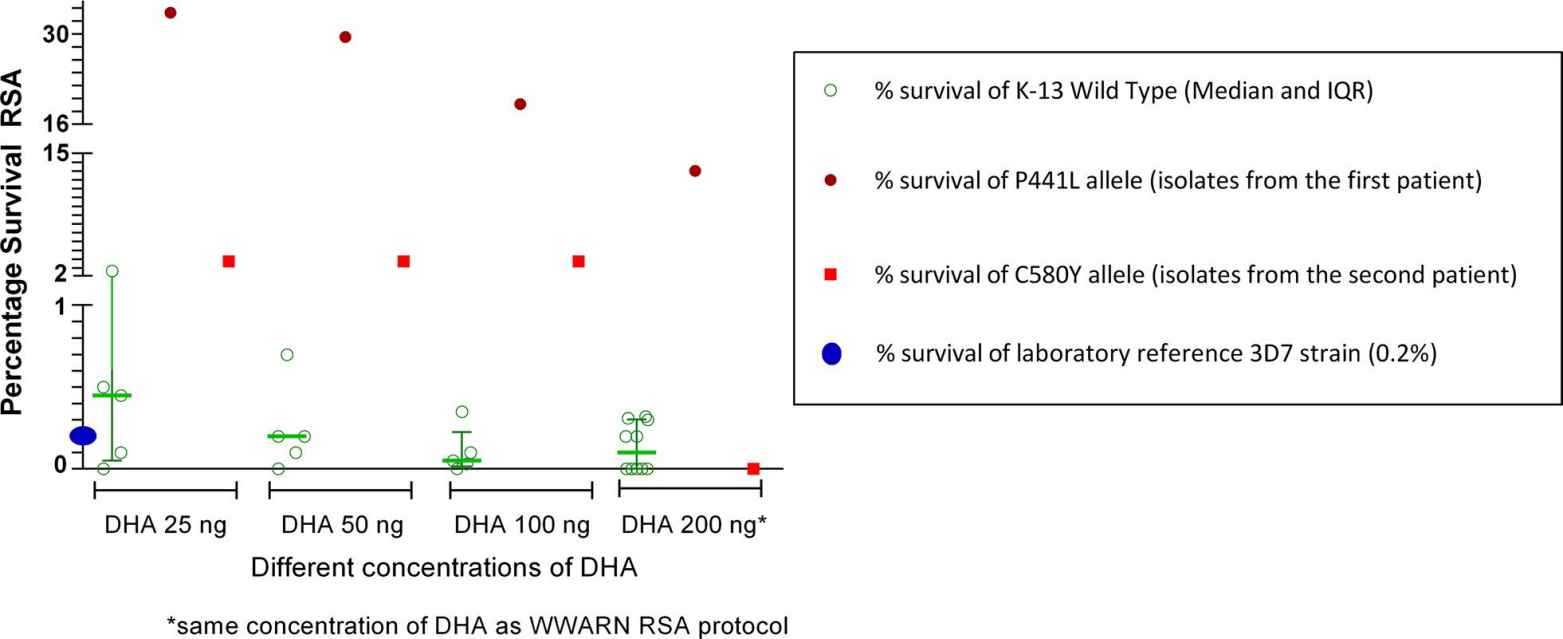
Percentage survival of *Plasmodium falciparum* isolates from two patients (red and brown) compared to laboratory reference 3D7 strain (blue dot) and Kelch-13 wild types (green) in in vitro Ring‐stage Survival Assay (RSA^0–3h^)

The in vitro Ring‐stage Survival Assay (RSA^0–3h^) was performed on the parasites obtained from these two patients (Fig. 2). In both cases in vitro assay confirmed that these isolates were resistant to artemisinin (see Methods in Additional file 2).

## Conclusions

Intravenous artesunate is the treatment of choice for severe malaria globally [3]. However, in this report, the parasitaemias in two patients with severe malaria treated with intravenous artesunate did not decline as expected and one patient died. Despite treatment, a proportion of patients will always die as a result of severe malaria. The presence of schizonts and neutrophils with malaria pigment [6] on the peripheral blood smear suggest that a significant proportion of the total parasite biomass was already undergoing sequestration. Artesunate and quinine may not have been able to reach these sequestered parasites. Similarly, the very high parasitaemia with slow parasite clearance in the second patient may have been partly attributable to his HIV status, since the development of opsonizing antibodies to variant surface antigens [10] and phagocytosis of opsonized infected erythrocytes by macrophages are impaired by HIV infection [11, 12]. Indeed, the time taken to clear the parasitaemia was far longer than usual following quinine monotherapy of presumed artemisinin-sensitive infections (parasite clearance in 83–96 h) [13, 14]. Nevertheless, there is uncertainty and clinicians treating patients with severe falciparum malaria acquired in areas where resistance to artemisinin is documented are confronted with difficult decisions when the parasite clearance fails to follow the expected decline.

The unique advantage of the artemisinins that makes them superior to other anti-malarials in treating severe malaria is their broad stage-specificity of action (especially on the early rings). This advantage is severely compromised by artemisinin resistance with implications for the treatment of severe malaria. A previous study of 69 patients conducted in 1998 [15] showed no improvement in parasite clearance from combining parenteral artesunate and quinine; however the safety of this combination was acceptable [15, 16]. The absence of an additive effect of artesunate and quinine at that time was explained by the broad-spectrum parasiticidal activity of artesunate, which results in very fast in vivo parasite clearance. This property is severely compromised in artemisinin-resistant infections.

In the absence of conclusive evidence, to minimize the risk to individual patients, it is recommended that all patients treated for severe malaria acquired in areas where artemisinin resistance is established should receive immediate empirical parenteral artesunate and quinine with frequent monitoring of parasite density.

## Additional files

**Additional file 1.** Parasite isolates collected after two doses of parenteral artesunate growing in drug-free plate.

**Additional file 2.** Methods and other table.
