# Supplementary figures and images for: Poor response to artesunate treatment in two patients with severe malaria on the Thai–Myanmar border
Source: Malar J. 2018 Jan 15;17:30. doi: 10.1186/s12936-018-2182-z (PMC5769511; doi:10.1186/s12936-018-2182-z)

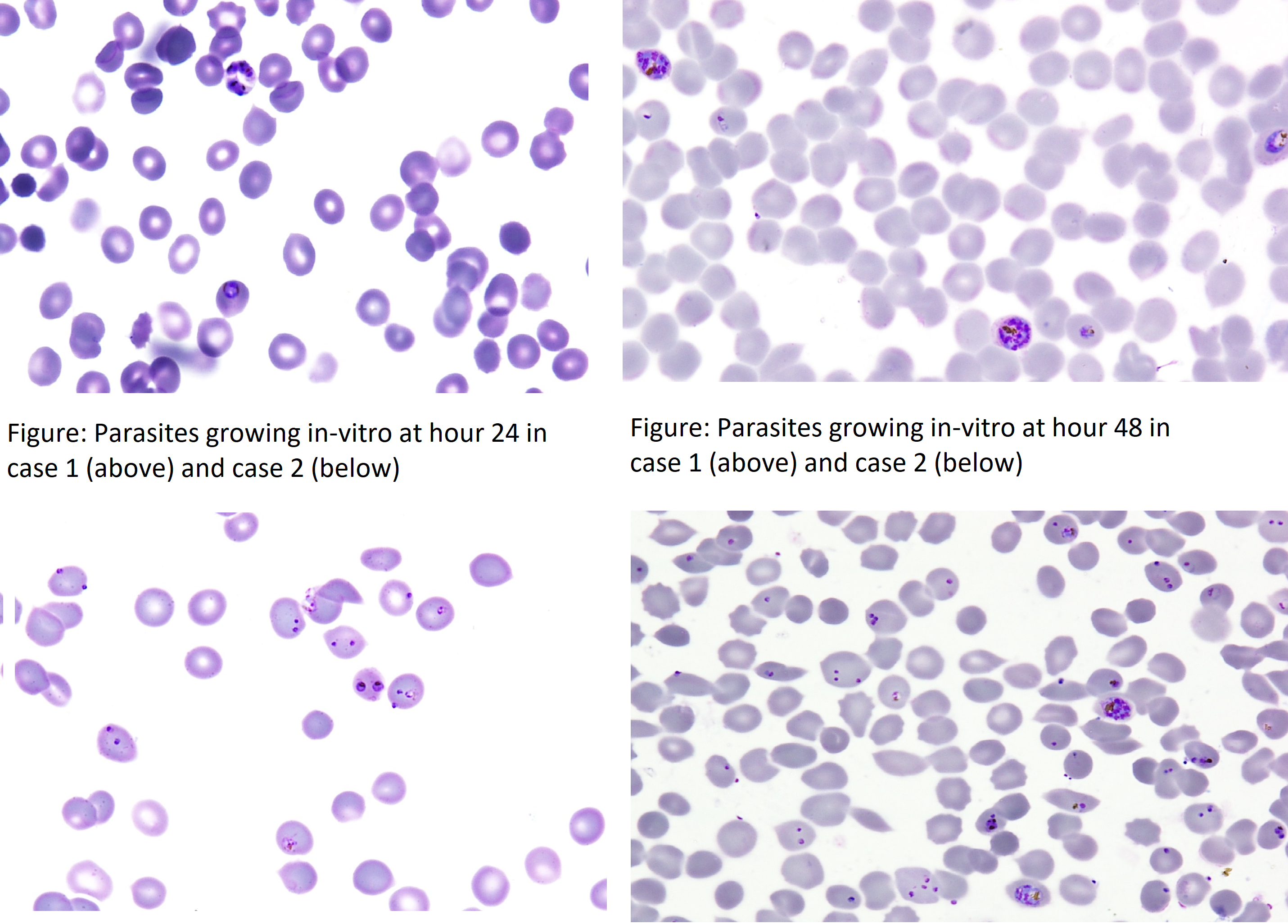

Supplement: Supplementary file 1 — Additional file 1. Parasite isolates collected after two doses of parenteral artesunate growing in drug-free plate. [file 12936_2018_2182_MOESM1_ESM.png]
