# Supplementary material for: Poor response to artesunate treatment in two patients with severe malaria on the Thai–Myanmar border
Source: Malar J. 2018 Jan 15;17:30. doi: 10.1186/s12936-018-2182-z (PMC5769511; doi:10.1186/s12936-018-2182-z)
Supplement: Supplementary file 2 — Additional file 2. Methods and other table. [file 12936_2018_2182_MOESM2_ESM.docx]

Additional Information

[List of abbreviations 2](#_Toc496604666)

[Measurement of drug concentrations 2](#_Toc496604667)

[In-vitro sensitivity assay 2](#_Toc496604668)

[Detailed parasitaemia results of both patients 3](#_Toc496604669)

[References for supplementary 4](#_Toc496604670)

## List of abbreviations

ACT Artemisinin-based combination therapy

ALP Alkaline Phosphatase

ALT Alanine aminotransferase

AST Aspartate aminotransferase

BUN Blood Urea Nitrogen

Hct Haematocrit

µL Microlitre

mmol/L Millimole per litre

PFT *Plasmodium falciparum* trophozoite

PFS *Plasmodium falciparum* schizont

PFG *Plasmodium falciparum* gametocyte

PRR Parasite Reduction Ratio

WWARN World Wide Antimalarial Resistance Network

WBC White blood cell

## Measurement of drug concentrations

Plasma drug concentrations were determined using an LC-MS/MS based assay, validated according to U.S. FDA guidelines [1]. In brief, solid-phase extraction (SPE) was used for the plasma sample preparation. The extracted drugs were separated using a Dionex Ultimate 3000 UHPLC (Thermo Fisher) equipped with a Zorbax SB-CN column (Agilent) or a Hypersil Gold C18 column (Thermo Fisher). An API500 triple-quadrupole mass spectrometer and Analyst 1.6.3 software (both ABSciex) were used for drug detection and quantification. The lower limit of quantification was 9.71 ng/mL for lumefantrine, 1.01 ng/mL for desbutyl-lumefantrine, 1.19 ng/mL for artesunate and 1.96 ng/mL for DHA. Internal standards were used to compensate for recovery and matrix effects and two replicates of quality control samples at low, middle, and high concentrations were included in the analysis to ensure precision and accuracy.

## In-vitro sensitivity assay

The in‐vitro Ring‐stage Survival Assay (RSA^0‐3h^) is performed to evaluate the susceptibility of *Plasmodium falciparum* to artemisinins. 0‐3-hour post‐invasion ring-stage parasites are exposed to different concentration of dihydroartemisinin (DHA) for 6 hours (200-100-50-25 ng) – approximating their drug exposure in patients treated with an artemisinin – and their survival is assessed 72 hours late. This assay used the same methodology as WWARN [2] except the modification in concentration of DHA (200 ng in WWARN vs. multiple concentration DHA including 200 ng as well). RSA^0‐3h^ survival rates from two patients at different DHA concentration are shown in comparison with K-13 wild type isolates and laboratory reference 3D7 isolate (Figure 2)

## Detailed parasitaemia results of both patients

Parasitaemia of case 1

| Hour | Parasitaemia per microlitre | Comments |
| --- | --- | --- |
| 0 | PFT 757368 PFS 352 PFG 1216 | >50% of PFT were late trophozoites. |
| 6 | PFT 746064 PFS 32 PFG 1824 | >80% of PFT were early ring. Intravenous quinine was added. |
| 12 | PFT 718432 PFS 16 PFG 1920 |  |
| 15 |  | Patient died. |

Parasitaemia of case 2

| Hour | Parasitaemia per microlitre | Comments |
| --- | --- | --- |
| 0 | PFT 1431840 PFS 11304 PFG 64 | 30-40% of PFT were late trophozoites. |
| 6 | PFT 2253264 PFS 3768 PFG 12 | 30-40% of PFT were late trophozoites. |
| 12 | PFT 1492379 PFS 352 PFG 32 | 30% of PFT were late trophozoites. Intravenous quinine was added. |
| 24 | PFT 1462988 PFS 144 PFG 128 | 30% of PFT were late trophozoites. Whole blood 350 ml was given because of very high parasitaemia and haematocrit dropped to 26%. |
| 36 | PFT 1632800 PFS 144 | 20% of PFT were late trophozoites. |
| 48 | PFT 664424 PFG 16 | 30% of PFT were late trophozoites. Whole blood 350 ml was given because of very high parasitaemia and haematocrit dropped to 23%. |
| 54 | PFT 606648 PFG 16 |  |
| 60 | PFT 678240 |  |
| 72 | PFT 1056296 | The parasitaemia had risen despite the patient recovered from the coma and started eating. So, atovaquone-proguanil combination was given orally to the patient. |
| 84 | PFT 678240 |  |
| 90 | PFT 203974 |  |
| 102 | PFT 101987 |  |
| 120 | PFT 18212 |  |
| 144 | PFT 1632 |  |
| Day 7 | PFT 160 |  |
| Day 8 | PFT 80 |  |
| Day 9 | PFT 32 |  |
| Day 10 | PFT 32 |  |
| Day 11 | PFT negative. |  |

## References for supplementary

1. Hanpithakpong, W, B Kamanikom, AM Dondorp, P Singhasivanon, NJ White, et al., *A liquid chromatographic-tandem mass spectrometric method for determination of artesunate and its metabolite dihydroartemisinin in human plasma.* Journal of Chromatography B, 2008. **876**(1): p. 61.

2. WWARN, WorldWide Antimalarial Resistance Network, *Ring-stage Survival Assays (RSA) to Evaluate the In-Vitro and Ex-Vivo Susceptibility of Plasmodium Falciparum to Artemisinins*. 2015.
